# Pneumothorax, Pneumomediastinum, Pneumoperitoneum, Pneumoretroperitoneum, and Cervical Emphysema After Colonoscopy: A Case Report

**DOI:** 10.7759/cureus.93896

**Published:** 2025-10-05

**Authors:** Cláudia Lima, Luísa Pereira, Inês Arnaud, Nuno Gonçalves, Teresa Almeida

**Affiliations:** 1 General Surgery, Unidade Local de Saúde do Alto Minho (ULSAM), Viana do Castelo, PRT

**Keywords:** colonoscopy, intestinal perforation, pneumomediastinum, pneumoperitoneum, pneumothorax, subcutaneous emphysema

## Abstract

Colonoscopy is the gold standard for the diagnosis and screening of colorectal diseases. Although uncommon, perforation is the most serious complication and may result in significant morbidity and mortality. We report the case of a 48-year-old woman who developed bilateral pneumothorax, pneumomediastinum, pneumoperitoneum, pneumoretroperitoneum, and cervical emphysema during therapeutic colonoscopy. The patient underwent bilateral chest tube placement and exploratory laparotomy, which revealed emphysematous infiltration of the cecal serosa but no free perforation. She recovered uneventfully after antibiotic therapy and supportive care. This case highlights the importance of maintaining a high index of suspicion for atypical presentations of colonoscopy-related complications and emphasizes that prompt recognition and timely intervention can be lifesaving.

## Introduction

Colonoscopy is widely used for both diagnostic and therapeutic purposes in a variety of colorectal diseases [1]. It is also the gold standard for colorectal cancer screening and for the evaluation of high-risk patients with a family history of polyps or carcinoma [2,3]. Although considered a safe procedure with a very low mortality rate (0.0029%) [4,5], colonoscopy is not without risks. Bleeding is the most common complication, whereas iatrogenic perforation, though rare, is the most serious and potentially life-threatening event, with reported morbidity of up to 36% and mortality rates around 7% [3,5].

The incidence of perforation ranges from 0.03% to 0.65% in diagnostic colonoscopies and may reach 0.07% to 2.14% in therapeutic procedures [1]. Several risk factors have been described, including advanced age (>65 years) and female sex [1,5]. In women, colonoscopy is considered technically more difficult due to a longer colon, deeper pelvis, and lower pain tolerance [4]. The presence of underlying bowel pathology, such as diverticular disease, tumors, or inflammatory bowel disease, also increases risk, as do previous radiotherapy, adhesions, and history of abdominal surgery [4,6]. The sigmoid colon is the most frequent site of perforation, followed by the cecum [5,6].

Most iatrogenic perforations are intraperitoneal, typically resulting in pneumoperitoneum and peritonitis [1]. However, if the defect occurs on the posterior wall of the ascending colon, descending colon, sigmoid colon, or rectum, the perforation may extend into the extraperitoneal space. In such cases, air may dissect into the retroperitoneum and spread through fascial planes to the mediastinum, pleural cavities, pericardium, and subcutaneous tissues, causing pneumoretroperitoneum, pneumomediastinum, pneumothorax, or subcutaneous emphysema [1]. Combined intra- and extraperitoneal perforations have also been reported [1]. Rare cases of extensive extraluminal air without visible perforation have also been described [2]. Diagnosis requires a high index of suspicion, particularly when clinical manifestations are atypical, such as dyspnea, hypoxemia, or subcutaneous emphysema rather than abdominal pain [1]. Management must be individualized and may include conservative treatment, endoscopic closure, surgical repair, or chest tube placement, depending on the clinical scenario and extent of injury [1].

Here, we present a case report of multi-compartment extraluminal air during colonoscopy after resection of two pedunculated polyps, followed by a review of the literature on this complication.

## Case presentation

We report the case of a 48-year-old woman with a history of normocytic normochromic anemia under investigation, who underwent colonoscopy for endoscopic resection of large colonic polyps. She had no other relevant medical history. The colonoscopy was performed under deep sedation with propofol, and the cecum was reached with adequate bowel preparation confirmed. The procedure was described as easy, with CO₂ insufflation used throughout.

In the distal ascending colon, a pedunculated (Paris 0-Ip) polyp measuring approximately 30 mm was identified and removed en bloc with a diathermic snare. The polypectomy site was inspected; no features of deep mural injury were seen (no target sign or visible muscularis propria). A single prophylactic metal clip was applied. In the mid-sigmoid colon, a pedunculated (Paris 0-Ip) polyp with a head measuring approximately 50 mm and a long, broad stalk was identified. An endoloop was placed at the base, producing cyanosis of the head. Multiple attempts at en bloc resection with a diathermic snare were unsuccessful, and the procedure became technically demanding and prolonged; the pedicle was therefore transected with a Flush-Knife. No immediate local complications were observed.

Following polyp resection, the patient developed oxygen desaturation and rapidly exhibited cervicothoracic and facial subcutaneous emphysema. No signs of perforation were observed in the sigmoid colon, and the procedure was immediately interrupted to stabilize the patient. Due to persistent desaturation, the anesthesiologist performed endotracheal intubation; the patient remained hemodynamically stable.

**Figure 1:**
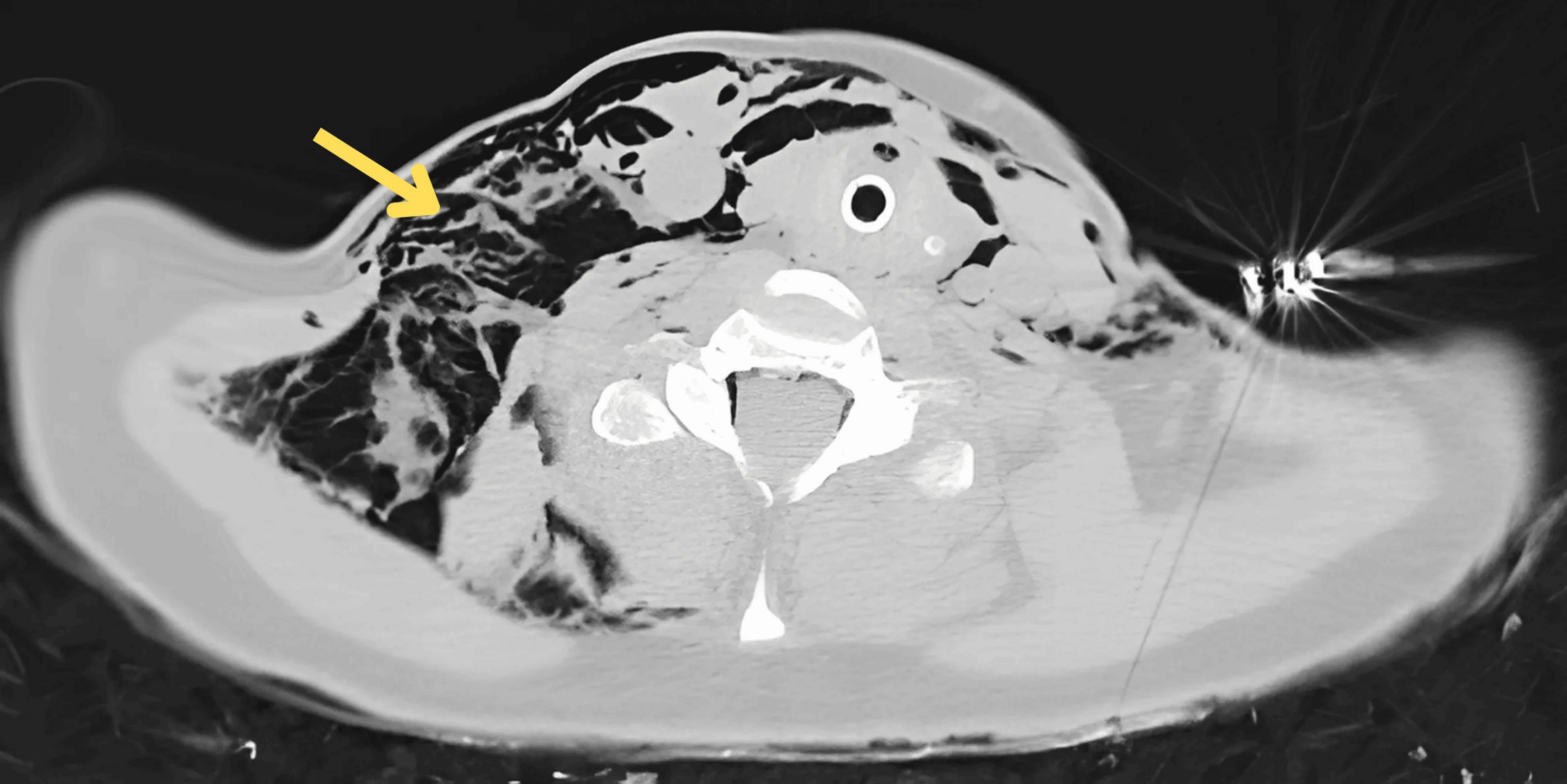
CT image (axial): cervical subcutaneous emphysema (arrow).

**Figure 2:**
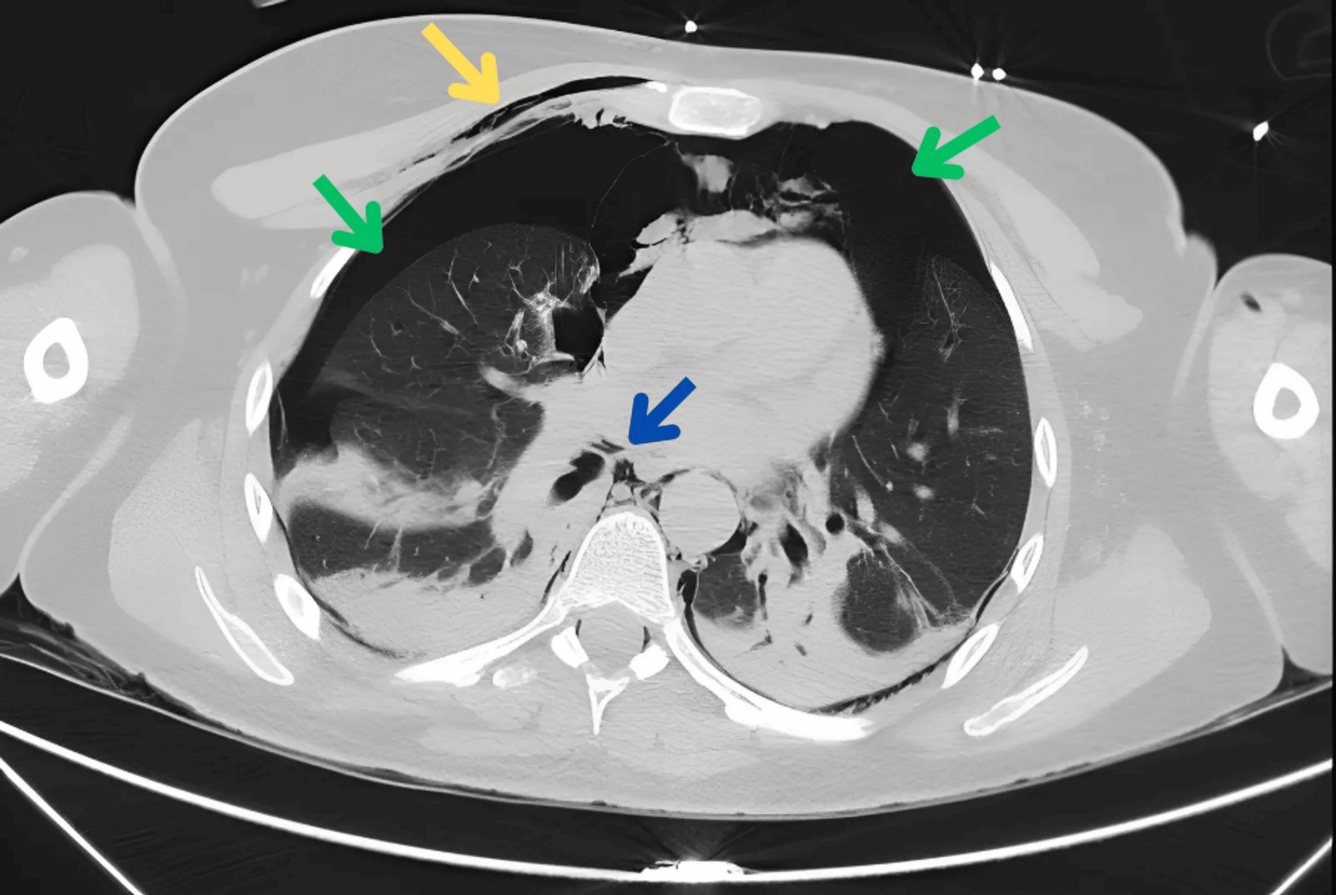
CT image (axial): bilateral pneumothorax. Blue arrow: pneumomediastinum; green arrow: pneumothorax; yellow arrow: subcutaneous emphysema

**Figure 3:**
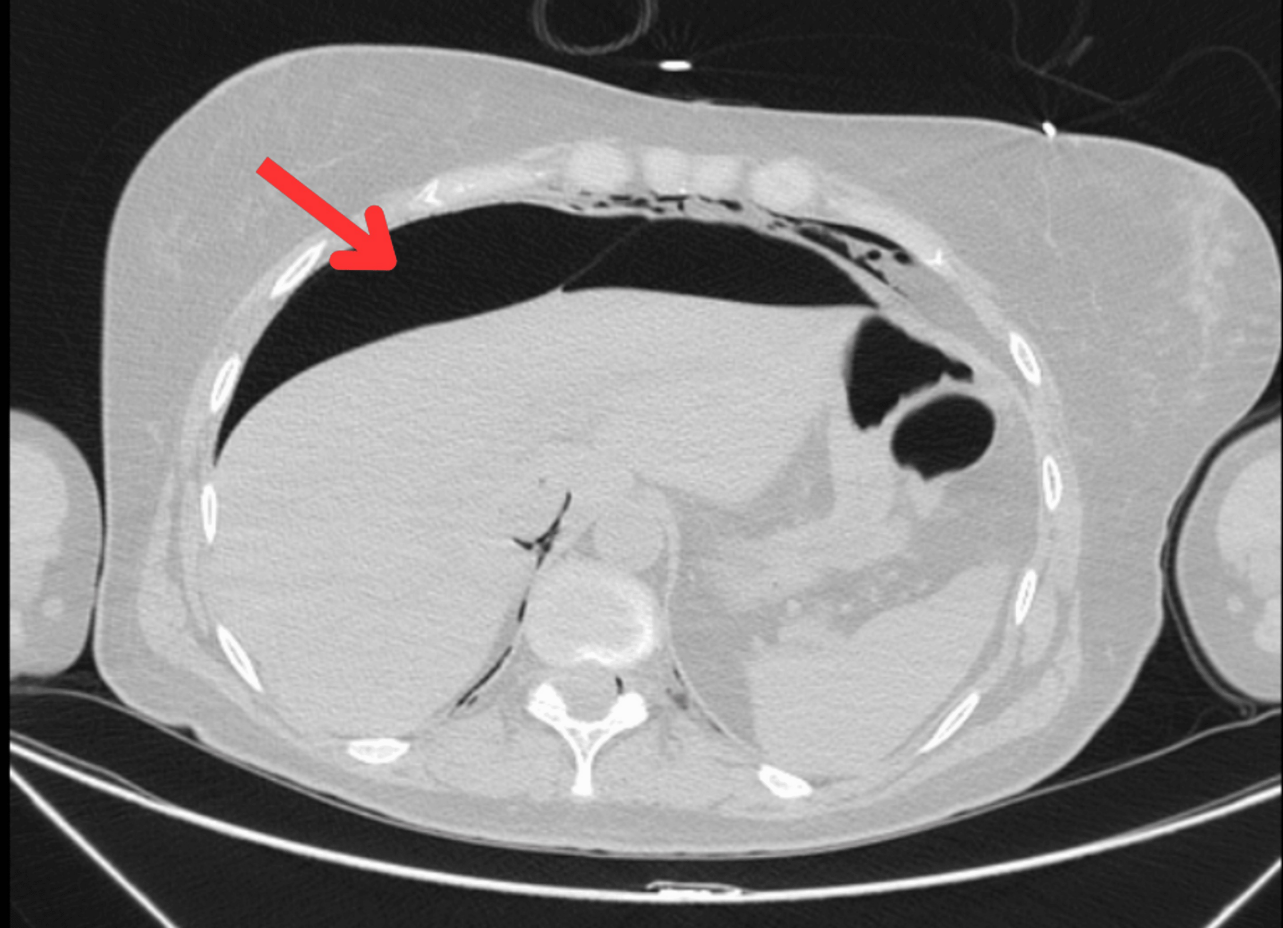
CT image (axial): pneumoperitoneum (arrow).

**Figure 4:**
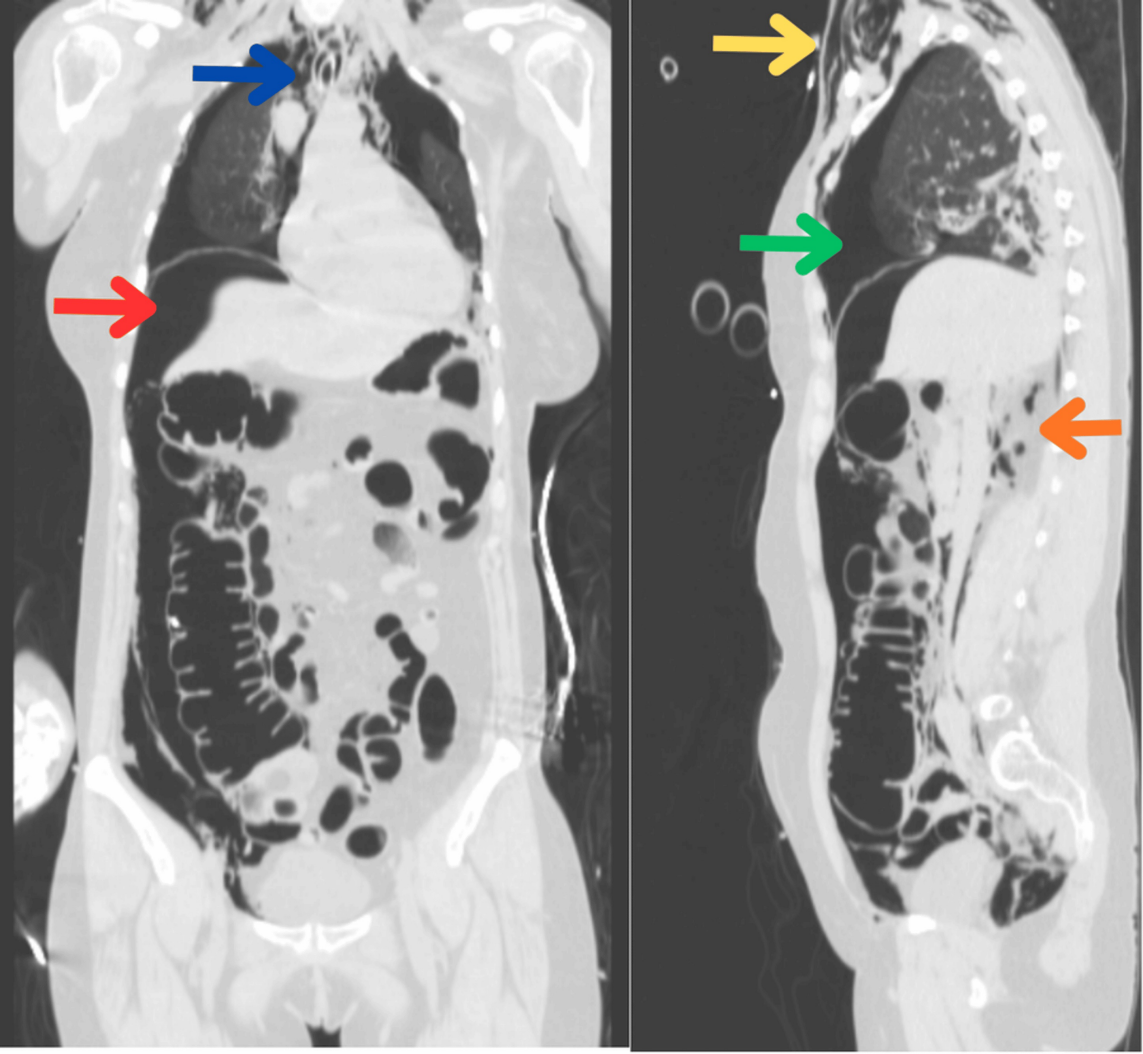
CT images: sagittal section (right) and coronal section (left). Blue arrow: pneumomediastinum; green arrow: pneumothorax; yellow arrow: subcutaneous emphysema; orange arrow: pneumoretroperitoneum; red arrow: pneumoperitoneum

A thoracoabdominopelvic computed tomography (CT) scan revealed bilateral pneumothorax, pneumomediastinum, pneumoperitoneum, pneumoretroperitoneum extending into the perirectal space, and extensive cervical, thoracic, and perianal subcutaneous emphysema (Figures 1-4).

**Figure 5:**
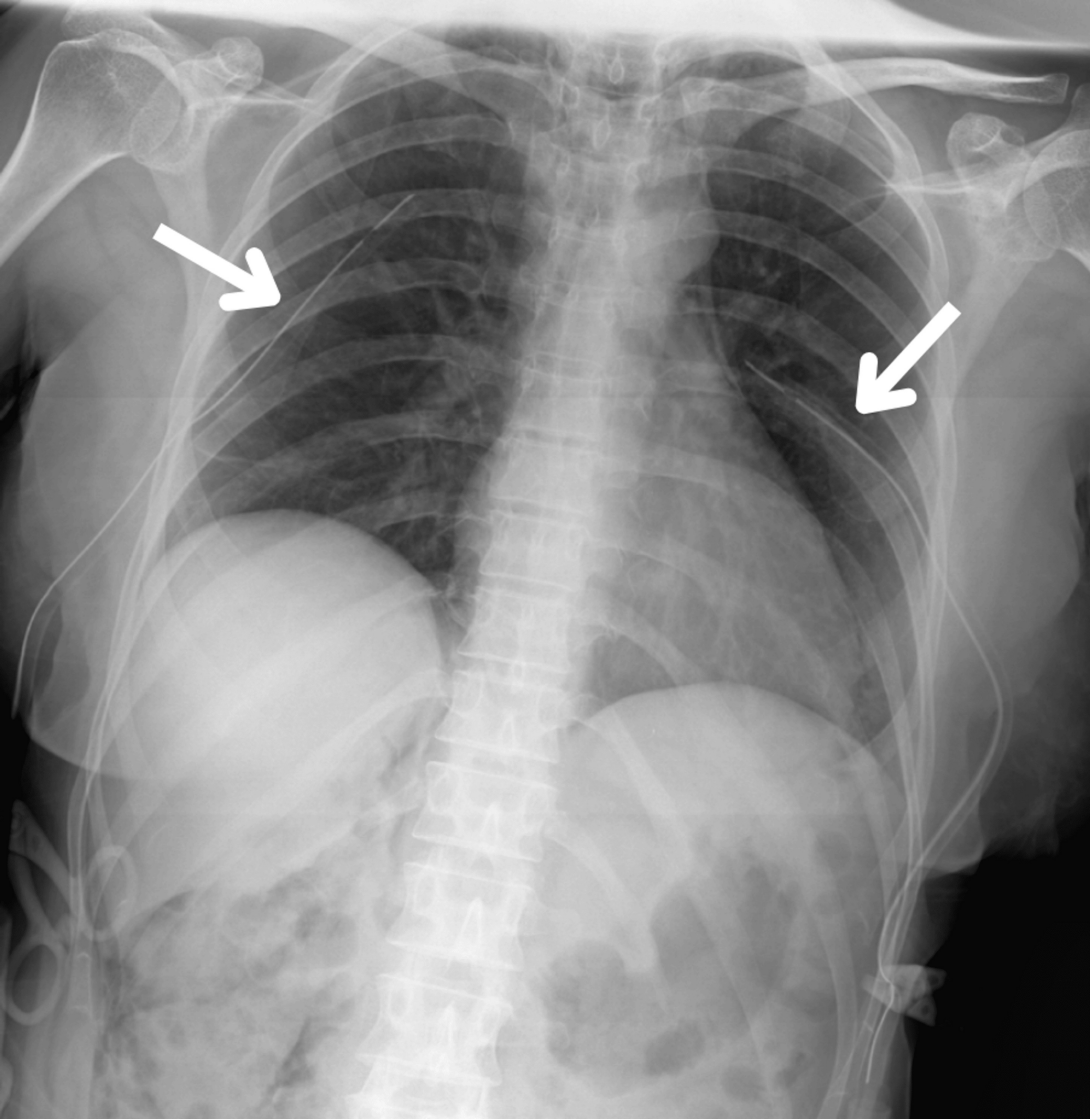
Chest radiography images after bilateral chest tube placement (arrows) with apparent resolution of bilateral pneumothorax.

The patient was transferred to the emergency department, where bilateral chest tubes were placed (Figure 5).

Laboratory results showed hemoglobin 9.4 g/dL, C-reactive protein <0.10 mg/dL, without leukocytosis or neutrophilia. The electrocardiogram was unremarkable.

**Figure 6:**
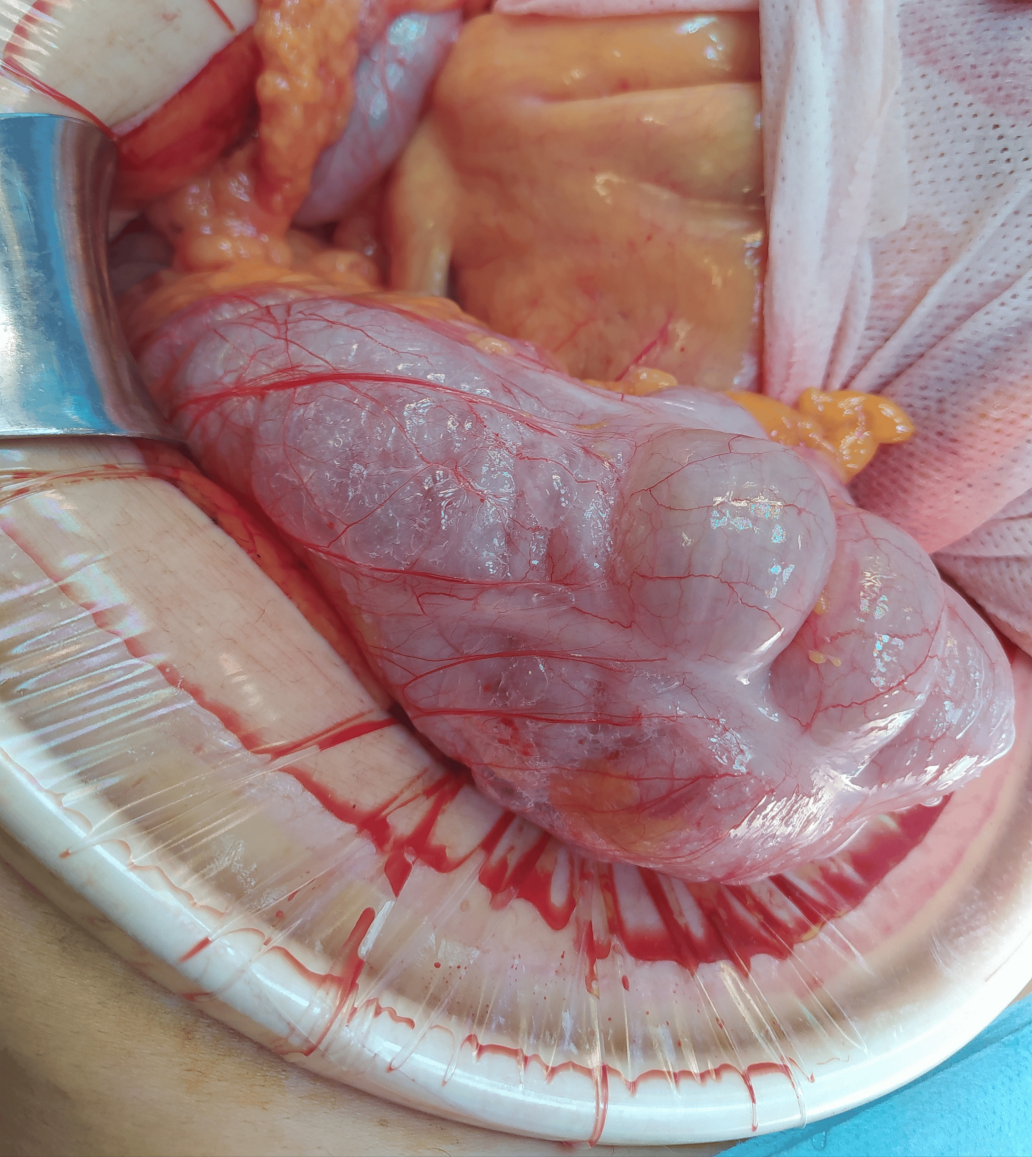
Intraoperative photograph. Emphysematous infiltration of the cecal serosa without gross perforation.

An exploratory laparotomy was then performed. Pneumoperitoneum was evident upon entry into the abdominal cavity, with distension of the cecum, ascending colon, and transverse colon. Emphysematous infiltration of the cecal serosa extending to the right paracolic gutter and hepatic flexure was observed (Figure 6). No free perforation, exudates, or ischemic changes were detected. The sigmoid colon appeared intact without emphysematous infiltration. Peritoneal lavage was performed, followed by closure of the abdominal wall.

The patient was admitted postoperatively to the intensive care unit (ICU) for monitoring and started on intravenous piperacillin-tazobactam. She showed rapid clinical improvement and was transferred to the surgical ward after 24 hours. Her recovery was uneventful, diet was progressively advanced, and she was discharged home on postoperative day seven.

## Discussion

Perforation is uncommon but remains the most serious colonoscopy complication; pneumothorax after colonoscopy is rarer still, largely limited to case reports and small series [1,4].

Perforations after colonoscopy can be categorized as (1) immediate, recognized during the procedure; (2) early post-procedural, diagnosed shortly after recovery; and (3) delayed, occurring ≥24 hours after an initially uneventful examination [7]. This distinction guides management: from endoscopic closure for recognized defects to risk-stratified observation versus operative intervention for early cases, with heightened vigilance for rare delayed thermal injuries.

The mechanisms responsible for intestinal perforation during colonoscopy include direct mechanical trauma, thermal injury, and barotrauma caused by excessive insufflation pressures [1,5]. Elevated pressures can separate the fibers of the muscularis propria, resulting in mucosal herniation. This herniated mucosa may either rupture, producing a free perforation, or become permeable to air, leading to extraluminal gas without a true defect [4]. Indeed, 5-15% of cases of post-colonoscopy pneumoperitoneum described in the literature occur without demonstrable perforation, as in our patient. In such cases, conservative management may be appropriate [1].

Several recognized risk factors present in our patient likely contributed to air dissection despite the absence of a gross defect: female sex, therapeutic resection of a large pedunculated polyp, and a technically demanding, prolonged second resection performed in the setting of a prior mucosal defect. Together, these factors increase the chance of microperforation or gas tracking along fascial planes during the procedure [1,4,7].

The most frequent symptom of post-colonoscopy perforation is abdominal pain [1]. Patients may also present with fever, leukocytosis, and tachycardia [6]. Clinical manifestations are not always immediate, since small perforations may be sealed by pericolic fat, omentum, or adjacent viscera [1]. When extraperitoneal perforation occurs, signs and symptoms can be atypical, including dyspnea, hypoxemia, or subcutaneous emphysema of the face, neck, or thorax [1]. In cases associated with pneumothorax, dyspnea is the most common presenting symptom [1]. Other rare clinical findings include pneumopericardium, pharyngeal or periorbital edema, and pneumoscrotum [6].

During a colonoscopy, our patient developed bilateral pneumothorax, pneumomediastinum, pneumoperitoneum, pneumoretroperitoneum, and cervical emphysema. These findings can be explained by the anatomical continuity of visceral spaces in the neck, thorax, and abdomen. The retroperitoneum, mediastinum, and cervical region communicate via fascial and perivascular planes, allowing the spread of insufflated air [1,4]. Pneumoretroperitoneum may result from direct retroperitoneal perforation or from air dissecting along the colonic wall (pneumatosis coli) and through the mesentery [8]. Pneumoperitoneum may arise from intraperitoneal perforation or decompression of retroperitoneal air into the peritoneal cavity [6]. Pneumothorax, in turn, can occur through rupture of the mediastinal pleura with decompression into the pleural cavity, or via diaphragmatic defects allowing translocation of peritoneal air [4].

Intraoperative findings, together with the pattern of extraluminal air, suggest a right-colonic source: either the post-polypectomy site or a de novo barotraumatic microperforation occurring during the technically demanding, prolonged second resection. In our case, no macroscopic perforation or contamination was identified, precluding definitive localization.

Although CO₂ insufflation facilitates faster gas absorption, it does not eliminate barotrauma or air dissection risk [7]. During technically demanding or prolonged resections (particularly when a prior polypectomy has left a mucosal defect), operators should monitor for progressive abdominal distension and any respiratory compromise, perform frequent exsufflation, and minimize insufflation to the least amount necessary. When feasible, staged resection, judicious use of cap-assisted/traction techniques, and early consideration of adjunctive closure (clip or loop-and-clip) for large stalk bases or vulnerable defects may further reduce the risk of leakage.

Management of colonoscopy-related perforation remains controversial due to the lack of randomized studies [1]. Treatment must be tailored to the patient’s clinical condition, the type and location of the lesion, the quality of bowel preparation, and the degree of contamination [1,4]. Conservative management can be considered in selected cases without peritonitis or evidence of intraperitoneal perforation [1,9]. Published literature on pneumothorax complicating colonoscopy consists mainly of isolated case reports and small series, with only a few dozen cases aggregated across reviews [4,9,10]. When respiratory compromise is severe, chest tube placement and sometimes endotracheal intubation are lifesaving [1]. Surgical options range from primary repair of the perforation site, segmental colectomy with anastomosis, to resection with stoma creation [1,4]. Endoscopic closure techniques may also be feasible in small, localized perforations [11].

This case underscores the importance of maintaining a high index of suspicion for extraperitoneal complications during colonoscopy. Prompt recognition and appropriate multidisciplinary management are critical to ensuring favorable outcomes.

## Conclusions

Pneumothorax after colonoscopy is an extremely rare but potentially life-threatening complication. Clinicians should maintain a high index of suspicion when patients develop dyspnea, oxygen desaturation, or subcutaneous emphysema during or shortly after the procedure. Prompt imaging with chest radiography or thoracoabdominopelvic CT is essential for diagnosis, and early intervention, particularly chest tube placement when indicated, can be lifesaving. This case also shows that extensive extraluminal air may occur without an identifiable perforation, reinforcing the need for individualized management and close post-procedural monitoring. Prevention hinges on using CO₂ insufflation (risk-reducing but not risk-eliminating), vigilant monitoring of abdominal distension, and frequent exsufflation, especially in difficult or prolonged resections or when a prior polypectomy has left a potential leak site.
